# Toward Synergies of Ketamine and Psychotherapy

**DOI:** 10.3389/fpsyg.2022.868103

**Published:** 2022-03-25

**Authors:** David S. Mathai, Victoria Mora, Albert Garcia-Romeu

**Affiliations:** ^1^Department of Psychiatry and Behavioral Sciences, Johns Hopkins University School of Medicine, Baltimore, MD, United States; ^2^School of Health Professions, Baylor College of Medicine, Houston, TX, United States

**Keywords:** ketamine, esketamine, therapy, psychotherapy, psychedelic, dissociation, ACT

## Abstract

Ketamine is a dissociative drug that has been used medically since the 1970s primarily as an anesthetic agent but also for various psychiatric applications. Anecdotal reports and clinical research suggest substantial potential for ketamine as a treatment in conjunction with psychological interventions. Here, we review historical and modern approaches to the use of ketamine with psychotherapy, discuss the clinical relevance of ketamine’s acute psychoactive effects, propose a unique model for using esketamine (one isomeric form of ketamine) with Acceptance and Commitment Therapy (ACT), and suggest considerations for moving medication-assisted psychotherapy forward as a field.

## Introduction

Ketamine emerged from the study of phencyclidine (PCP) derivatives suitable for anesthetic use in humans, and its discovery in 1962 is attributed to Parke-Davis Labs and Dr. Calvin Lee Stevens, a professor of organic chemistry at Wayne State University (Domino et al., 1965). Early clinicians recognized ketamine’s dreamlike and hallucinogenic properties, experienced by patients as feeling “disconnected” from their environment, and leading to its classification as a “dissociative anesthetic” (Domino et al., 1965; Reier, 1971). The subjective effects associated with ketamine were observed to be “pleasant” (Domino et al., 1965), and investigators soon began to consider its broader value as an antidepressant and psychotherapeutic agent (Yensen, 1973; Fontana and Loschi, 1974; Sofia and Harakal, 1975). The drug has since been explored for a growing number of psychiatric applications both as a standalone medication and in combination with supportive interventions.

A compelling and transdiagnostic body of clinical research has emphasized the use of ketamine in combination with psychotherapy for mental health conditions. While psychotherapy is not easily defined, it has been reasonably described as an interpersonal intervention that relies on repeated encounters, a healing relationship, and a particular explanatory model, all within a structured therapeutic frame (Greenway et al., 2020). Psychotherapy may be simplified even further, to denote any “communication between patients and therapists that is intended to help people (American Psychological Association, 2017).” These definitions will be used hereafter to discuss a range of psychotherapeutic modalities involving ketamine administration.

The idea of harnessing psychotherapy with pharmacotherapy has been considered before. The additive and synergistic effects of combining conventional antidepressants and psychotherapy, for example, are well-established (Cuijpers et al., 2014; Dunlop, 2016). Some precedent even exists for the approval of drug-therapy combinations by regulatory bodies such as the U.S. Food and Drug Administration [FDA; 21 CFR 201.57 (c)(2)(i)(A), 2022]. Medications such as naltrexone extended-release injectable suspension for alcohol and opioid dependence, bupropion hydrochloride extended-release for smoking cessation, and buprenorphine sublingual tablets for opioid dependence are all labeled for use in conjunction with therapy. This is also expected to be the case if psilocybin and MDMA are approved as psychiatric treatments for major depression and post-traumatic stress disorder (PTSD), respectively (Yazar-Klosinski and Mithoefer, 2017; Johnson et al., 2018).

Combination strategies serve a variety of purposes, driven largely by the limitations of single-modality interventions. These strategies serve to address treatment non-response, residual symptoms, and relapse and recurrence involving psychiatric illness (Dunlop, 2016). Others have further emphasized that approaches to pharmacotherapy that include psychotherapy have the potential to improve psychological flexibility, quality of life, and overall functioning, beyond symptom reduction, in ways that are valuable to patients (Kamenov et al., 2017; Watts and Luoma, 2020). Integrated approaches may also promote drug safety and tolerability. For instance, a therapy-oriented approach to psilocybin administration in research settings is thought to mitigate the psychological risks that are possible with treatment (Johnson et al., 2008). The use of psychotherapy with ketamine could similarly address treatment risks, for example, by allowing for drug-sparing treatment paradigms that are of public interest and decrease the likelihood of tachyphylaxis (Mathai et al., 2020, 2021).

What is perhaps most unique to the use of ketamine as a therapeutic adjunct is its status as a highly psychoactive, medically legal, and reliably perplexing agent. Here, we review historical and modern approaches to the use of ketamine with psychotherapy, discuss the clinical relevance of ketamine’s acute psychoactive effects, propose a unique model for working with esketamine (the S-enantiomer of ketamine), and make suggestions for moving forward as a field. We emphasize a conceptual understanding of ketamine and psychotherapy, rather than the specific parameters of treatment administration.

## A Brief History

The first known uses of ketamine as a therapeutic adjunct date back to the early 1970s, soon after its approval as an anesthetic agent. In Mexico, the psychiatrist Salvador Roquet discovered that subanesthetic doses of the drug occasioned mental states that could be combined with psychoanalytical techniques and indigenous healing practices in an approach he called “psychosynthesis” (Yensen, 1973; Wolfson, 2014). His pioneering and controversial work incorporated the use of multiple psychedelic substances and “sensory overload” in group treatment settings with the goal of producing, and then processing, extreme psychological experiences. Through this method, Roquet believed that patients could confront existing psychological conflicts and achieve emotional catharsis.

Around the same time, physicians in Southern Iran were exploring similar qualities of ketamine in the individual treatment of hospitalized psychiatric patients (Khorramzadeh and Lotfy, 1973). Enayat Khorramzadeh and Atta Ollah Lofty observed that ketamine facilitated patients’ ability to engage in an “abreactive” psychotherapy process involving the recollection and processing of traumatic memories, which was ultimately associated with enduring relief of depression, anxiety, and other psychiatric symptoms (see Table 1 for characteristics of this and other research trials involving ketamine and psychotherapy). They later conducted a follow-up study examining how dimensions of personality contributed to the psychoactive effects of ketamine and found that elements of “extraversion,” “neuroticism,” and “psychoticism” could reliably predict drug experiences, suggesting the importance of nonpharmacological factors in treatment (Khorramzadeh and Lotfy, 1976).

**Table 1:** Characteristics of studies reviewed using ketamine and psychotherapy.

Study	N	Design	Treatment population	Drug parameters	Psychotherapy parameters
Adams et al. (2017)	1	Case study	Refractory obsessive compulsive disorder	50 mg IN ketamine; twice weekly for 4 weeks	16-week program of inpatient/outpatient ERP; for inpatient weeks 3–6, therapy was accompanied by twice-weekly administration of ketamine; unclear timing of therapy relative to ketamine administrations
Azhari et al. (2021)	8	Uncontrolled trial	Cannabis use disorder	1–2 IV ketamine infusions over 4 weeks; Infusion 1: (0.71 mg/kg over 52 min) on week 2; Infusion 2 (non-responders): (1.41 mg/kg over 92 min) on week 4	6-week program of MET and MBRP with therapy occurring outside of ketamine administrations (i.e., MET therapy on the day before the infusion and the afternoon of the infusion)
Dakwar et al. (2019)	55	Randomized controlled trial	Cocaine use disorder	Treatment group: Single IV ketamine infusion (0.5 mg/kg over 40 min); Control group: Single IV midazolam infusion (0.025 mg/kg over 40 min); infusions on day 2	5-week inpatient/outpatient program of MBRP; 1 MBPR session daily during first 5 days; followed by 8 sessions of MBRP outpatient (twice-weekly for 4 weeks); Patients received IV infusion on day 2 of inpatient stay; therapy occurred outside of ketamine/ midazolam administration (i.e., MBRP 2 h post-infusion)
Dakwar et al. (2020)	40	Randomized controlled trial	Alcohol use disorder	Treatment group: Single IV ketamine infusion (0.71 mg/kg over 52 min); Control group: Single IV midazolam infusion (0.025 mg/kg over 52 min); infusions on week 2	5-week outpatient program of MET; 6 sessions of MET over 5 weeks (1 session/ week); therapy occurred outside of ketamine/ midazolam administration (i.e., MET session provided 24 h after infusion)
Dames et al. (2022)	94	Observational study	Mixed	3 sessions of PO or IM ketamine (during weeks 4, 5, and 7): either PO or IM ketamine for session 1 (dose unspecified); IM ketamine (1–1.5 mg/kg) for sessions 2 and 3	12-week treatment program including group meetings and KaT with model of preparation, dosing, and integration; therapy occurred outside of ketamine administration (i.e., “initial sharing” began after 90 min, and post-KaT group integration sessions occurred within 36 h)
Dore et al. (2019)	235	Observational study	Mixed	SL, IM, (or both) ketamine; Average dose range was 200–250 mg SL, and 80–90 mg IM	Outpatient KAP with sessions typically 2 weeks apart, or more frequently depending on acuity; number of sessions ranged from 1–25, which were spread over variable time periods from initial session, to visit evaluation, to termination where applicable; therapy occurred before, during and after ketamine administrations
Grabski et al. (2022)	96	Randomized controlled trial	Alcohol use disorder	Treatment group: 3 weekly IV ketamine infusions (0.8 mg/kg over 40 min); Control group: 3 saline infusions; Infusions occurred at visits 2, 4, and 6 spaced 1–3 weeks apart	Treatment group: 7 sessions of MBRP; Control group: 7 sessions of AE; therapy began at visit 2 and continued for the subsequent six visits; therapy occurred outside of ketamine administration (i.e., infusion was always preceded by MBRP or AE and followed by another session about 24 h later)
Halstead et al. (2021)	1	Case study	Persistent depressive disorder and treatment-resistant post-traumatic stress disorder	SL ketamine (150 mg); 4 administrations over 13 days	13-day intensive outpatient therapy program consisting of MBCT and FAP; therapy occurred before, during and after ketamine administrations
Khorramzadeh and Lofty (1973)	100	Non-randomized controlled trial	Mixed	IV ketamine infusions in 3 dose ranges: (1) 0.2–0.3 mg/kg; (2) 0.4–0.6 mg/kg; (3) 0.7–1.0 mg/kg; unknown duration of infusion	“Abreactive” psychotherapy during drug administration
Krupitsky et al. (1992)	186	Randomized controlled trial	Alcohol use disorder	Treatment group: Single co-administration of aethimizol (1.5% 3 ml, IM), bemegride (0.5% 10 ml IV), and ketamine (3 mg/kg, IM); Control group: Conventional aversive therapy without ketamine administration	ACA method of alcoholism treatment with therapy occurring before, during, and after ketamine administration
Krupitsky and Grinenko (1997)	111	Non-randomized controlled trial	Alcohol use disorder	Treatment group: Single co-administration of aethimizol (1.5% 3 ml, IM), bemegride (0.5% 10 ml, IV), and ketamine (2.5 mg/kg IM); Control group: Conventional therapy for alcoholism without ketamine	KPT method of treatment with therapy occurring before, during, and after ketamine administration
Krupitsky et al. (2002)	70	Randomized controlled trial	Heroin use disorder	Treatment group: Single IM ketamine injection (2 mg/kg: hallucinogenic dose); Control group: Single IM ketamine injection (0.2 mg/kg: non-hallucinogenic dose)	KPT method of treatment with therapy occurring before, during, and after ketamine administration
Krupitsky et al. (2007)	59	Randomized controlled trial	Heroin use disorder	Treatment group: 3 IM ketamine injections (2 mg/kg); 1-month intervals between doses; Control group: Single IM ketamine injection (2 mg/kg)	Addiction counseling and KPT method of treatment with therapy occurring before, during, and after ketamine administration
Ocker et al. (2020)	1	Case study	Opioid medication dependence with opioid-induced hyperalgesia	5-day (inpatient) continuous IV infusion of ketamine in combination with a multimodal analgesia regimen; ketamine dose titrated throughout admission (0.09–0.59 mg/kg/h)	Outpatient CBT every 3–4 weeks after initial period of ketamine administration
Pradhan et al. (2018)	20	Randomized controlled trial	Treatment-refractory post-traumatic stress disorder	Treatment group: Single IV infusion of ketamine (0.5 mg/kg over 40 min); Control group: Single IV infusion of normal saline over 40 min	12 sessions of TIMBER; therapy occurred during and then after single ketamine administration
Ragnhildstveit et al. (2021)	1	Case study	Treatment-resistant bulimia nervosa	18 IV ketamine infusions (0.5 mg/kg IV over 40 min) over 3-month period	18 sessions of guided psychotherapy during ketamine administrations and preceded by 30 min of preparatory psychotherapy
Rodriguez et al. (2016)	10	Uncontrolled trial	Obsessive compulsive disorder	Single IV infusion of ketamine (0.5 mg/kg over 40 min)	10 sessions of ERP over 2-week period; therapy occurred outside of ketamine administration (i.e., after completion of single ketamine administration)
Sappington et al. (1979)	21	Randomized controlled trial	Healthy volunteers	Treatment group: IV ketamine infusion (0.1 mg/lb); Control groups: No ketamine	“Induced-anxiety” therapy focused on induction of negative affect prior to drug administrations, drug-induced relaxation, and processing with therapist
Shiroma et al. (2020)	12	Uncontrolled trial	Chronic, moderate post-traumatic stress disorder	3 IV infusions of ketamine (0.5 mg/kg over 40 min); once weekly for the first 3 weeks of treatment; unclear if IV infusions continued after week 3	10-week program of PE with therapy occurring during ketamine administrations
Wilkinson et al. (2017)	16	Uncontrolled trial	Major depressive disorder	4 IV infusions of ketamine (0.5 mg/kg over 40 min) over 2 weeks	10-week program of CBT with therapy occurring outside of ketamine administrations
Wilkinson et al. (2021)	41	Randomized controlled trial	Severe major depressive disorder and treatment-resistant depression	6 IV infusions of ketamine (0.5 mg/kg over 40 min) over 3 weeks	Treatment group: 14-week program of CBT; Control group: 14-week program of TAU; therapy occurred outside of ketamine administrations

*ACA, affective contra-attribution; AE, alcohol education; CBT, cognitive behavioral therapy; ERP, exposure response prevention; FAP, functional analytic psychotherapy; IM, intramuscular; IN, intranasal; IV, intravenous; KaT, ketamine assisted therapy; KAP, ketamine assisted psychotherapy; kg, kilograms; KPT, ketamine assisted psychotherapy; lb, pounds; MBCT, mindfulness based cognitive behavioral therapy; MBRP, mindfulness based relapse prevention therapy; MET, motivational enhancement therapy; mg, milligrams; min, minutes; PO, per os; TAU, treatment as usual; TIMBER, trauma interventions using mindfulness based extinction and reconsolidation*.

Several other significant explorations of ketamine as a therapeutic agent occurred through the 1970s and 1980s (Lilly, 1972, 1978; Fontana and Loschi, 1974; Sappington et al., 1979; Grof, 1980; Golechha et al., 1985), though the single most comprehensive body of clinical research in this area can be credited to the Russian physician, Evgeny Krupitsky. Krupitsky first used ketamine in a form of behavioral psychotherapy in the former Soviet Union in 1985 (Kolp et al., 2014). In his earliest studies, ketamine was combined with other agents to induce unpleasant psychedelic experiences that were associatively linked with alcohol toward the goal of decreasing alcohol use (Krupitsky et al., 1992; Sivolap and Savchenkov, 1994). Krupitsky eventually realized that patients benefited similarly from positive, transcendent experiences while on ketamine and shifted from a model of aversive conditioning to one informed by existential and transpersonal psychology. This model has been described as Ketamine Psychedelic Psychotherapy (KPP) or Ketamine Psychedelic Therapy (KPT), and was used successfully in the treatment of alcohol and opioid use disorders (Krupitsky and Grinenko, 1997; Krupitsky et al., 2002, 2007). Moreover, these studies indicated that ketamine could be combined with psychotherapeutic interventions to produce meaningful and enduring changes in psychological attitudes, concepts of self, and overall functioning. Krupitsky’s research was ultimately shuttered by the rescheduling of ketamine in Russia amidst growing concerns around its recreational use (Kolp et al., 2014).

## Modern Approaches

Widespread psychiatric interest in ketamine accelerated with positive findings from the first randomized controlled trial (RCT; Berman et al., 2000) and a larger replication study (Zarate et al., 2006) of ketamine as a standalone treatment for depression. These and most subsequent academic investigations have assumed a “biochemical paradigm” (Bennett, 2019), wherein the therapeutic benefits of ketamine are attributed to a pharmacologic effect independent of perceived psychoactivity or supporting interventions. This paradigm is, for example, evident in current FDA-approved uses of esketamine as an antidepressive and antisuicidal agent (McIntyre et al., 2021).

However, in the mid-2000s, it became apparent that some community practitioners were continuing to work with ketamine as had been done historically, using it as a tool to facilitate psychological exploration and healing (Kolp et al., 2006, 2007; Early, 2014; Ring et al., 2016). A new wave of ketamine-assisted psychotherapy (KAP) began to emerge, attracting a growing network of clinicians and informed theoretically by principles of psychedelic therapy (Dore et al., 2019; Halstead et al., 2021; Ragnhildstveit et al., 2021; Dames et al., 2022). As with other psychedelic therapies, KAP emphasizes attention to “set and setting” (Dore et al., 2019) – a broad conceptualization of the nonpharmacological parameters that are thought to shape hallucinogenic drug response, such as the degree of preparation before, and the environment during, drug administrations (Hartogsohn, 2016). Administrations of ketamine within KAP follow “a dosage escalation strategy to achieve different levels of trance increasing to full out-of-body experiences” while holding central “that some degree of mind alteration is necessary for ketamine’s effects (Dore et al., 2019).” This framework suggests the possibility of highly variable and dose-dependent states of consciousness induced by ketamine that represent different opportunities for therapeutic intervention (Kolp et al., 2014; Dore et al., 2019). For example, the KAP context would be compatible with both “psycholytic therapy,” typically involving lower doses of psychoactive drug to facilitate the therapeutic and relational quality of ongoing psychotherapy during acute drug effects, and “psychedelic therapy,” involving higher doses of drug to facilitate the occurrence and integration of profound, mystical- and peak-type experiences (Garcia-Romeu and Richards, 2018). KAP, along with early uses of ketamine in psychiatry, can then be classified as “experience-oriented” approaches to treatment, for which the subjective quality of drug effect is thought to have inherent value and be an integral part of the therapeutic process (Mathai et al., 2020). In this framework, the patient’s experiences under drug effects can be considered meaningful and potentially insightful material, which can then be utilized in collaboration with the therapist to facilitate therapeutic progress.

**Figure 1:**
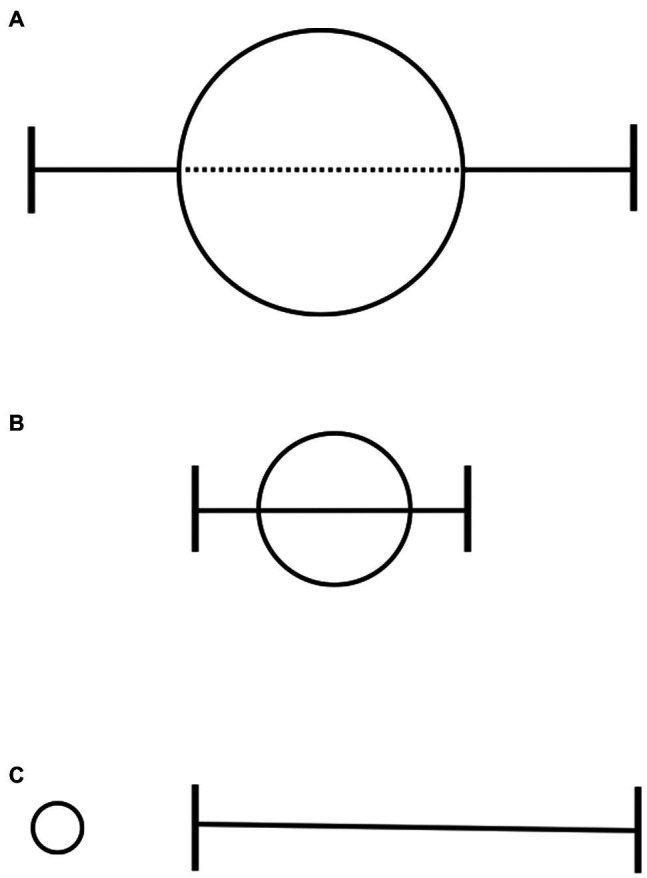
Functional units for existing models combining ketamine with psychotherapy. Each circle indicates a single experience of ketamine, with circle size corresponding to relative theoretical emphasis on the nature of the experience. Bracketed lines are used to indicate optimal windows of psychotherapeutic intervention. **(A)** shows the strategy of high-dose “psychedelic therapy,” involving significant preparation before and integration after a limited number of drug sessions, which themselves are largely inner-directed, rather than primarily relational, experiences. **(B)** shows the model of low-dose “psycholytic therapy,” in which ongoing psychotherapy coincides with ketamine administration, making use of acute drug effects that are thought to facilitate the quality of therapy. The two former approaches are examples of “experience-oriented” frameworks, such as KAP. **(C)** is representative of “plasticity-oriented” strategies, wherein psychotherapy is delivered after the period of acute drug effects but within “critical periods” of neural adaptation that are thought to facilitate the uptake and efficacy of behavioral interventions.

Other recent and contrasting approaches have tended to use behavioral therapies largely outside of the period of acute drug effects (Rodriguez et al., 2016; Adams et al., 2017; Wilkinson et al., 2017, 2021; Dakwar et al., 2019, 2020; Ocker et al., 2020; Shiroma et al., 2020; Azhari et al., 2021; Philipp-Muller et al., 2021; Grabski et al., 2022). This strategy presumes that, beyond the period of its immediate psychoactive function, ketamine produces a window of enhanced neuroplasticity and other neural adaptations that facilitate cognitive and behavioral interventions (Dakwar and Nunes, 2016; Wilkinson et al., 2019; Hasler, 2020). Theoretically, drug-facilitated psychotherapy could be optimized when delivered during “critical periods” of neural development, marked by exquisite sensitivity to environmental input and potentially conducive to learning (Lepow et al., 2021). It could be argued that this “plasticity-oriented” approach is rooted most in the function of ketamine as a “psychoplastogen” – a term used to describe small molecule neurotherapeutics that produce rapid and measurable changes in plasticity after a single administration that are thought to support relatively long-lasting changes in behavior (Olson, 2018). This logic suggests the possibility of ketamine-like psychoplastogens that might enhance psychotherapeutic processes in critical periods after drug administration without the need for marked mind-altering effects (Olson, 2021). Consistent with this understanding, predominantly plasticity-oriented approaches to ketamine as a therapeutic adjunct differ from KAP in how psychological support is allocated, with relatively less emphasis on or engagement with drug-induced experiences over the course of treatment (see Figure 1 for illustration of these models).

The “experience-oriented” and “plasticity-oriented” uses of ketamine outlined above could overlap but may be seen as portrayals of two leading theories of treatment. Though not described here, other nuanced psychotherapeutic applications of ketamine are emerging (Pradhan et al., 2018; Veen et al., 2018; Bottemanne et al., 2021; Bottemanne and Arnould, 2021; Muscat et al., 2021b), and novel ways of using the drug will continue to develop. For all approaches, there exist scientific questions that can engage key theoretical assumptions. For example, how do animal models of critical periods and adaptive learning inform the timing and nature of complex, idiosyncratic interventions using ketamine in humans? This research is still in its infancy. Additionally, and of particular relevance to clinicians, what is the role of the subjective ketamine experience in ketamine therapy? Current findings on this point are reviewed below.

## The Relevance of Experience

It has been argued that no study to date has demonstrated a therapeutic effect for ketamine absent some degree of perceived psychoactivity (Dore et al., 2019). Several challenges are inherent to such an undertaking, including the experimental prospect of defining “psychoactivity,” which would seemingly include experiences ranging from feeling “high,” “relaxed,” “connected,” “happy” and “light” to the peak- and mystical-type phenomena that occur at sufficiently high doses of ketamine. A “critical test” of this question has been proposed (Yaden and Griffiths, 2021), wherein a psychedelic agent demonstrates full and lasting therapeutic efficacy when administered to individuals while unconscious and who subsequently report no memory of drug-related experience. In counterpoint, Olson (2021) draws attention to several interesting, albeit limited, studies suggesting that intraoperative ketamine may improve mood in surgical patients even when they are unconscious during drug administration (Kudoh et al., 2002; Jiang et al., 2016; Xu et al., 2017). Among issues with poor generalizability due to experimental design, it is unclear if the demonstrated effects from these studies meet criteria for “full and lasting therapeutic efficacy.” More suitable investigations of the “critical test” are underway (Heifets, 2021), but in the meantime, other types of correlational data can provide insight into the relationship between subjective drug experience and therapeutic efficacy.

The acute ketamine effect for which there is the most data is “dissociation,” as measured by the Clinician-Administered Dissociative States Scale (CADSS) and includes feelings such as detachment from oneself and one’s environment and changes in sensory perception (Grabski et al., 2020). Several independent reviews have identified experimental evidence of a positive correlation between dissociation and antidepressant efficacy for ketamine, although this relationship has been inconsistent when examined across clinical trials (Ballard and Zarate, 2020; Grabski et al., 2020; Mathai et al., 2020). Another large post-hoc analysis of phase 3 clinical trial data found no mediating effect of dissociation on the antidepressant effects of esketamine (Chen et al., 2022). While these findings are difficult to interpret, it is notable that modern studies have classified dissociation primarily as an adverse event, suggesting that a major limitation in the predictive ability of the CADSS, and similarly utilized instruments, is prevailing research bias toward these psychoactive effects as undesirable. Most studies of ketamine have also not examined the relevance of dissociation in the context of psychotherapy.

It is nonetheless worth considering that dissociation, as a single metric and captured by the CADSS, may not be a useful predictor of the therapeutic efficacy of ketamine. Other subjective effects during drug administration, including ratings of “happiness” (Chen et al., 2020) or the sensation of “lightness” (Stocker et al., 2019) have also been associated with antidepressant benefit. Conversely, anxiety experienced during ketamine infusions appears to predict negative treatment response for depression (Aust et al., 2019). These findings require replication but together suggest that the affective quality of drug-induced experience may also be pertinent to therapeutic outcomes with ketamine. It is moreover possible that the benefits of certain subjective effects, such as drug-mediated increases in connectedness (Kolp et al., 2014; Griffiths et al., 2021; Mollaahmetoglu et al., 2021), are not fully realized in the absence of interpersonal therapeutic engagement.

Finally, it is valuable to recognize the similarities between ketamine and classic (serotonergic) psychedelics such as psilocybin and lysergic acid diethylamide (LSD), which produce overlapping subjective phenomena in spite of diverging pharmacological mechanisms of action (Bowdle et al., 1998; Studerus et al., 2010). For classic psychedelics, it is well-established that a subset of psychoactive effects, often referred to as mystical-type effects and characterized by a sense of unity, predict greater therapeutic response across a variety of conditions such as depression, existential distress, and substance use disorders (Garcia-Romeu et al., 2014; Griffiths et al., 2016; Roseman et al., 2018). Interestingly, it has been hypothesized that the same mechanisms that drive the efficacy of these treatments may also be responsible for dose-dependent psychiatric risks, like that of psychosis (Haarsma et al., 2021). However, the relevant psychoactive effects of classic psychedelics appear to be optimized in careful experimental conditions that consider the benefit, safety, and tolerability of such (Johnson et al., 2008; dos Santos et al., 2018). While similar optimization has not been pursued for ketamine, increasing research suggests that mystical- and peak-type experiences, such as measured by the Hood Mysticism Scale (HMS) and 11D-ASC questionnaire, increase the likelihood of various therapeutic benefits (Dakwar et al., 2014, 2018; Mollaahmetoglu et al., 2021; Rothberg et al., 2021; Sumner et al., 2021). Regardless of whether these effects are essential to treatment, there is mounting support for a broader understanding of and attention to the spectrum of experiences induced by ketamine.

## A Specific Proposal

In light of existing knowledge gaps, the authors and colleagues have proposed a pilot investigation of esketamine with Acceptance and Commitment Therapy (ACT) for treatment-resistant depression that is currently in the planning stage. ACT has been recognized as a well-suited framework for psychedelic-assisted treatment (Walsh and Thiessen, 2018; Sloshower et al., 2020; Wolff et al., 2020). ACT is a form of cognitive-behavioral therapy that emphasizes psychological flexibility (PF) and has been shown to be effective in the treatment of depressive symptoms (Bai et al., 2020). PF is a transdiagnostic construct that can be thought of as an individual’s capacity to recognize and adapt to contextual demands, shift mindset or behaviors during individual and social experiences, maintain balance across important life domains, and to be aware of and committed to behaviors consistent with values (Kashdan, 2010). PF appears to predict outcomes of psychotherapy for treatment-resistant depression (Yasinski et al., 2020) and measurements of this construct, like The Acceptance and Action Questionnaire (AAQ-II), have been shown to mediate the relationship between acute psychedelic effects and subsequent decreases in depression and anxiety (Close et al., 2020; Davis et al., 2020). Importantly, it is also held that the quality and therapeutic value of relevant psychedelic effects are influenced by the specific treatment context for classic psychedelics (Johnson et al., 2008; Hartogsohn, 2016; Carhart-Harris et al., 2018; Garcia-Romeu and Richards, 2018), although this has not been adequately explored with ketamine. Taken together, these data indicate that PF-oriented models may be key to supporting processes of change when psychotherapy and psychedelic experience are combined (Watts and Luoma, 2020).

While several published studies indicate the value of psychotherapy alongside ketamine administration, none of these investigations have utilized an ACT-based approach. Furthermore, to our knowledge no studies have examined the combination of esketamine and concurrent psychotherapy. In this pilot study we propose to examine three research questions: (1) will an augmented esketamine treatment protocol (AET) involving preparatory counseling and ongoing ACT during esketamine administration yield greater or more durable antidepressant-type effects than treatment as usual (TAU) esketamine dosing? (2) will the AET treatment context produce significantly different subjective (e.g., dissociative-type) effects than TAU esketamine dosing? and (3) Are esketamine-induced subjective effects within either treatment context associated with antidepressant efficacy?

**Figure 2:**
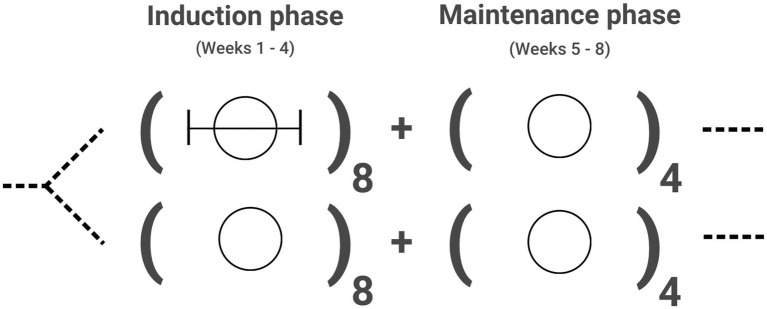
Proposed design for a pilot investigation of esketamine with Acceptance and Commitment Therapy (ACT) for treatment-resistant depression. Current esketamine dosing protocols call for twice weekly dosing during a 4-week induction phase, followed by weekly dosing during a maintenance phase in weeks 5 through 8. Patients receive an initial dose of 56 mg, followed by repeated doses of 56 mg or 84 mg based on treatment response (i.e., efficacy and tolerability). After initial study procedures, participants will be randomized to receive treatment as usual (plain circle) or esketamine in conjunction with ACT (circle with bracketed line) during the induction phase of treatment. Both study arms will follow the same clinical procedures for maintenance treatment and subsequent follow-up.

The study will randomly assign patients initiating esketamine for treatment-resistant depression to either the TAU or AET arm beginning with the first dose and throughout the duration of the 4-week induction phase (see Figure 2). The fact that the FDA requires a two-hour monitoring window after esketamine dosing provides a unique opportunity for psychotherapy that coincides with the period of acute drug effects. ACT sessions will be conducted approximately 1 h after drug administration, and we expect that standard dosages of esketamine will facilitate a “psycholytic” and relational model of therapy. Participant treatment response will be tracked using standard measures of depression from baseline through the end of the 4-week maintenance period, and again at a 3-month follow-up. Subjective effects will be assessed throughout esketamine dosing using a battery of psychometric questionnaires. Other measurements of interest include psychological flexibility, therapeutic alliance, and need for maintenance treatment that may differ between treatment arms. These data will provide needed empirical observations regarding the impact of psychotherapeutic intervention in conjunction with esketamine, and the mediating role of subjective and contextually determined drug effects.

## Discussion

Ongoing study will help clarify the uses of ketamine as a therapeutic adjunct. Notably, in contrast to pharmacotherapy development, no formal approval process exists for specific psychotherapies, which are ultimately defined as “evidence-based” when research involving a substantial number of patients has provided evidence of treatment effect (National Institute of Mental Health, 2021). However, these lines of inquiry are unlikely to attract the funding of pharmaceutical companies and will thus depend heavily on the support of public agencies (Migone, 2017). Furthermore, with the need for time-consuming and complex factorial study designs to adequately compare drug x therapy interactions (Grabski et al., 2022), standards of care for combinations of ketamine and psychotherapy are expected to develop slowly.

For the time being, there is value to considering existing and independent medical and psychological standards. Prescribing of ketamine, particularly for off-label uses that may have therapeutic utility, should be based on “firm scientific rationale and on sound medical evidence” (U.S. Food and Drug Administration, 1998), such as effectiveness data collected from controlled trials or documented in clinical settings (Radley et al., 2006). Best psychological practice is informed not only by existing research but also “clinical expertise in the context of patient characteristics, culture and preferences (American Psychological Association, 2008).” Additional parameters are expected to be relevant to the interactions of ketamine and therapy, including variables such as medication dosage and the timing of psychological interventions, as described here, along with other factors, such as the normative claims of treatment, and provider training in specific therapeutic modalities.

Different paradigms for ketamine can coexist – the “experience-oriented,” “plasticity-oriented,” and others still. Even guidelines for strictly pharmacological uses of ketamine and esketamine emphasize the importance of “a comfortable and adaptable environment” for patients given that drug administration “may amplify sensory experiences and/or result in dissociation or psychotomimetic effects (McIntyre et al., 2021).” An integrated approach to ketamine therapy, considering both historical perspectives on subjective experience and modern advances in neuroscience, may ultimately lead to a better understanding of relevant drug effects, improved treatment protocols, and multiple dimensions of benefit (Muscat et al., 2021a; Walsh et al., 2021). Much like the “abreactive” interventions of old, ongoing applications of ketamine are expected to reflect the prevailing psychological zeitgeist. However, while the mechanics, contexts, and explanations for our interventions may shift, the therapeutic endeavor is the same as it has always been – toward the vision of a better life for patients.

## Data Availability Statement

The original contributions presented in the study are included in the article/supplementary material, further inquiries can be directed to the corresponding author.

## Author Contributions

DM, VM, and AG-R made substantial contributions to the conception, design, and drafting of the manuscript. All authors approved the final version of this manuscript and agree to be accountable for all aspects of the work.

## Funding

Funding for this research was made possible by the Johns Hopkins Center for Psychedelic and Consciousness Research and provided by Tim Ferriss, Matt Mullenweg, Blake Mycoskie, Craig Nerenberg, and the Steven and Alexandra Cohen Foundation. The funders had no role in the study design, data collection and analysis, decision to publish, or preparation of the manuscript.

## Conflict of Interest

AG-R receives support from the Heffter Research Institute and serves as a scientific advisor to ETHA Natural Botanicals and NeonMind Biosciences.

The remaining authors declare that the research was conducted in the absence of any commercial or financial relationships that could be construed as a potential conflict of interest.

## Publisher’s Note

All claims expressed in this article are solely those of the authors and do not necessarily represent those of their affiliated organizations, or those of the publisher, the editors and the reviewers. Any product that may be evaluated in this article, or claim that may be made by its manufacturer, is not guaranteed or endorsed by the publisher.
